# Expanding the genomic diversity of human anelloviruses

**DOI:** 10.1093/ve/veaf002

**Published:** 2025-01-07

**Authors:** Sejal Modha, Joseph Hughes, Richard J Orton, Spyros Lytras

**Affiliations:** MRC-University of Glasgow Centre for Virus Research, The University of Glasgow, Glasgow G61 1QH, United Kingdom; MRC-University of Glasgow Centre for Virus Research, The University of Glasgow, Glasgow G61 1QH, United Kingdom; MRC-University of Glasgow Centre for Virus Research, The University of Glasgow, Glasgow G61 1QH, United Kingdom; MRC-University of Glasgow Centre for Virus Research, The University of Glasgow, Glasgow G61 1QH, United Kingdom; Division of Systems Virology, Department of Microbiology and Immunology, The Institute of Medical Science, The University of Tokyo, Tokyo 108-0071, Japan

**Keywords:** genomic diversity, evolution, human virus, anellovirus, metagenomics

## Abstract

Anelloviruses are a group of small, circular, single-stranded DNA viruses that are found ubiquitously across mammalian hosts. Here, we explored a large number of publicly available human microbiome datasets and retrieved a total of 829 anellovirus genomes, substantially expanding the known diversity of these viruses. The majority of new genomes fall within the three major human anellovirus genera: *Alphatorquevirus, Betatorquevirus*, and *Gammatorquevirus*, while we also present new genomes of the under-sampled *Hetorquevirus, Memtorquevirus*, and *Samektorquevirus* genera. We performed recombination analysis and show evidence of extensive recombination across all human anelloviruses. Interestingly, more than 95% of the detected events are between members of the same genus and only 15 inter-genus recombination events were detected. The breakpoints of recombination cluster in hotspots at the ends and outside of the ORF1 gene, while a recombination coldspot was detected within the gene. Our analysis suggests that anellovirus evolution is governed by homologous recombination; however, events between distant viruses or ones producing chimaeric ORF1s likely lead to nonviable recombinants. The large number of genomes further allowed us to examine how essential genomic features vary across anelloviruses. These include functional domains in the ORF1 protein and the nucleotide motif of the replication loop region, required for the viruses’ rolling-circle replication. A subset of the genomes assembled in both this and previous studies are completely lacking these essential elements, opening up the possibility that anellovirus intracellular populations contain nonstandard viral genomes. However, low-read depth of the metagenomically assembled contigs may partly explain the lack of some features. Overall, our study highlights key features of anellovirus genomics and evolution, a largely understudied group of viruses whose potential in virus-based therapeutics is recently being explored.

## Introduction

Advances in high-throughput sequencing technologies and reduced sequencing costs have had a strong impact on virus research specifically in the context of virus discovery (Wolf et al. 2020). Viruses are the most abundant entities in our ecosystem, and they are found in all environments captured and catalogued by researchers (Edwards and Rohwer 2005, Nayfach et al. 2021). Metaviromic approaches are less biased compared to traditional culture-based techniques and have enabled the discovery of a large, and still expanding, diversity of previously unknown viruses (Edgar et al. 2022).

Anelloviruses are small, circular, single-stranded DNA (ssDNA) viruses with negative-sense genomes that range from 1.6 to 3.9 kb in length (Miyata et al. 1999, Varsani et al. 2021). The first identified anellovirus, named torque teno virus (TTV), was found in 1997 in the blood sample of a hepatitis patient (Nishizawa et al. 1997). Since then, investigators hypothesized the potential involvement of these viruses in disease, partly because of their prevalence in immunocompromised patients and blood transfusion samples (Simmonds et al. 1998, Itoh et al. 2000). However, it now seems that anelloviruses are not pathogenic and are likely commensals instead, ubiquitously found in virtually all individuals (Cossart 2000, Vasilyev et al. 2009, Koonin et al. 2021). For years, the nonpathogenic nature of these viruses has made them fall outside of the focus of mainstream virology research and many aspects of their genomics, diversity, and overall biology remain poorly understood. The recent push for ramping up virus discovery through metagenomics and the potential use of anelloviruses in viral-based therapeutics has reignited the interest in anellovirus research (Arze et al. 2021, Liou et al. 2022, Butkovic et al. 2023).

In the last few years, a large diversity of anelloviruses has been catalogued in a range of studies led by Moustafa et al. (2017), Cebriá-Mendoza et al. (2021), Tisza et al. (2020), Arze et al. (2021), Laubscher et al. (2022), and Laubscher et al. (2023). These genomes have been found not only in closely monitored blood transfusion patients but also in metagenomic datasets from many other types of samples including faeces, nasal secretions, saliva, urine, and bile, suggesting their omnipresence in all types of human microbiomes and nonspecific tissue tropism (Kaczorowska and Van Der Hoek 2020). Their presence is not unique to humans, with diverse anelloviruses having been discovered in many other mammals (Okamoto et al. 2000, Abe et al. 2000; Okamoto et al. 2001, Fahsbender et al. 2017, Kraberger et al. 2021a). There is currently a total of 173 species classified in 34 genera under the *Anelloviridae* family, according to the International Committee on Taxonomy of Viruses (ICTV) Master Species List v39.3 (ratified in June 2024). The higher taxonomic placement of the family has been ambiguous until recently due to the viruses’ genetic distinctness to other circular DNA virus groups. However, Butkovic et al. (2023) propose that anelloviruses are classified into a new phylum, the ‘Commensaviricota’, under the *Shotokuvirae* kingdom (realm *Monodnaviria*).

The human-infecting members of the family are classified into four main genera: *Alpha- torquevirus* (TTV), *Betatorquevirus* (TT mini viruses—TTMV), *Gammatorquevirus* (TT midi viruses—TTMDV), and *Hetorquevirus* (Varsani et al. 2021). Three additional human-associated genera were recently accepted by the ICTV, the: *Memtoquevirus, Samektorquevirus*, and *Lamedtorquevirus* (Laubscher et al. 2023). The three key genera of human anelloviruses that contain the vast majority of known members have different genome lengths. TTVs have the largest genomes that range from 3.6 to 3.9 kb, TTMVs have genomes that range between 2.8 and 2.9 kb, whereas TTMDVs have a slightly wider genome size range from 2 to 3.2 kb (Taxonomy of Viruses, International Committee on 2011). All anellovirus genomes code for one major open reading frames (ORFs), referred to as ORF1. Some genomes also contain few partially overlapping accessory ORFs, but their function is less well-characterized and will not be analysed in this paper (Kaczorowska and Van Der Hoek 2020). ORF1 is used for species and genus delineation and has recently been confirmed to function as the capsid protein of the virion, containing a jelly-roll-like domain and forming a capsid of 60 ORF1 monomers in an icosahedral (T = 1) conformation (Liou et al. 2022, Butkovic et al. 2023). Another distinct feature of the ORF1 is an arginine (and lysine)-rich, N-terminal sequence motif (ARM) that localizes within the capsid polymer. This positively charged part of the protein likely stabilizes the genome when inside the virion and may be involved in genome replication and packaging (Liou et al. 2022). Like most circular DNA viruses, anelloviruses replicate their genome through rolling-circle replication. The noncoding part of the genome contains a small (eight nucleotide), conserved sequence motif, surrounded by GC-rich flanking regions, which is likely cleaved to act as the origin of replication (Villiers et al. 2011). Lastly, much of the sequence diversity observed across the anelloviruses may be caused by extensive recombination, taking place during the viruses’ replication (Worobey 2000, Arze et al. 2021, Kraberger et al. 2021b).

In a previous study by Modha et al. (2022), UnXplore—a systematic framework to identify, assemble, and quantify unknown sequences present in various human microbiomes—was developed and applied to a range of human microbiome datasets. Modha et al. (2022) examined 40 different studies covering 963 samples across 10 human microbiomes, such as faecal, oral, and skin, and found that 2% of sequences remain unclassified, indicating a vast potential for discovering new viruses. In this study, we extend these findings to mine sequences categorized as known and partially known in Modha et al. (2022). Specifically, we analyse, identify, and focus on the diversity of anelloviruses present in the publicly available human microbiome datasets (*n* = 2596) that were investigated using UnXplore (Modha et al. 2022), expanding our understanding of the anelloviruses circulating in humans. We shed light on the phylogenetic relatedness between the human anellovirus genera, their overall patterns of genomic recombination, and the distribution of essential features in their genomes.

## Methods

### Metagenomic assembly and virus sequence identification

A total of 61 studies spanning, 11 human microbiomes (bodily sites/sample types) were analysed as part of a bigger project looking for signatures of unknown sequences in human microbiome samples using the UnXplore framework developed by (Modha et al. 2022). Briefly, the UnXplore framework is an unknown sequence identification and quantification framework that can assemble high-quality contigs from human microbiome datasets; although focussed on ‘unknown’ sequences, the framework identifies those of known origin at the same time. We utilized the samples analysed as part of (Modha et al. 2022) (*n* = 963) as well as an additional 2596 human blood microbiome datasets retrieved from the Sequence Read Archive (SRA) repositories. Each sample was *de novo* assembled and investigated for the presence of unknown sequences (often referred to as biological ‘dark matter’) using the UnXplore framework.

The contig set generated from the UnXplore analysis was consolidated and filtered to remove short contigs that were <1-kb long. A total of 7,196,090 contigs were then submitted to three separate virus prediction tools: VirSorter2, DeepVirFinder (Ren et al. 2020, Guo et al. 2021) and TetraPredX (https://github.com/sejmodha/TetraPredX). The prediction results were filtered using the following criteria. For DeepVirFinder, contigs with a score ≥0.9 and *P*-value < .05 were selected, for VirSorter2 all predicted contigs with a minimum score 0.5 were selected and for TetraPredX all contigs with viral signal probability ≥.95 and all other class probability ≤0.5 were selected. These contigs were then searched against an extensive set of nucleotide (nt) and protein sequence (refseq_protein) databases for further validation. The top 25 hits for each contig were extracted and the Lowest Common Ancestor (LCA) was computed from the sequence similarity results obtained using BLAST (nucleotide) (Camacho et al. 2009) and DIAMOND (protein level) (Buchfink et al. 2021). Based on the LCA, each contig’s superkingdom was retrieved from the NCBI taxonomy database using ete3 (Huerta-Cepas et al. 2016). A proportion of virus hits and subsequently virus families were determined for each contig using the ‘ExtractLCA.py’ and ‘ExtractLCABLASTM6.py’ scripts included in UnXplore.

In total, 272,827 contigs of interest (with LCA root or viruses) were examined which included a set of 122,884 confirmed virus contigs. From this set, a total of 2005 contigs were confirmed and matched exclusively to anelloviruses and were extracted. This set included contigs that were 1000–8220 bases long. From this set, contigs that were shorter than 2-kb (*n* = 709) and longer than 4 kb (*n* = 19) were removed based on the known length of anellovirus genomes. The remaining 1277 contigs that were between 2 and 4-kb long were retained and used for all subsequent analyses described in this study.

To validate the assembly of contigs from sample SRR2037085 from which we retrieved an unusually large number of anellovirus genomes, we also assembled the reads in this sample with MEGAHIT (Dinghua et al. 2015), a different assembler made specifically for metagenomic assembly. We extracted MEGAHIT contigs with lengths of more than 300 nt and used BLAST (Camacho et al. 2009) to compare them to the SRR2037085 contigs assembled from our original pipeline. We confirm that 269 contigs assembled with MEGAHIT had matches to the reference contigs with at least 90% coverage.

### Processing of virus genomes

To obtain a set of representative sequences, 1277 anellovirus contigs were clustered using MMseqs2 easy-cluster pipeline with—min-seq-id 0.95,—cov-mode 1-c 0.99 parameters (Steinegger and Söding 2017), and a set of 840 representative sequences was obtained. To ensure that an ORF1 protein is encoded by each of the sequences, we used the EMBOSS getorf application (Rice et al. 2000) with a minimum size of 1000 nt and assuming that the sequences are circular. We could not identify an ORF1 using these criteria for 11 sequences which were excluded in downstream phylogenetic analysis, resulting in a final set of 829 novel anellovirus genomes. The genomic completeness of each contig was assessed using the viralComplete pipeline with a coverage threshold of 100 (https://github.com/ablab/viralComplete/), indicating 220 complete genomes and 609 near-complete/partial genomes.

All 829 anellovirus contigs have been annotated using the Prokka annotation software (Seemann 2014) and are publicly available in the Third Party Annotation Section of the DDBJ/ENA/GenBank databases under the accession numbers TPA: BK068993-BK069821, as well as at https://doi.org/10.5281/zenodo.14408301.

To complement the novel genomes presented in this study with the available anellovirus diversity, we retrieved all human anellovirus genome sequences discovered in a study led by Tisza et al. (2020) (BioProject: PRJNA396064), as well as those analysed in a recent study led by Varsani et al. (2021). Getorf was also used in these sequences, as above, to identify the coordinates of the ORF1 coding region. All genomes were then rotated so that the ORF1 start codon was at the start of the sequence to enable downstream sequence aligning. Genome sequences with no identifiable ORF1 coding sequence were excluded.

Additionally, we retrieved all ORF1 coding sequences from the SCANellome anellovirus database described in Laubscher et al. (2023) consisting of all sequences available in NCBI Genbank (https://github.com/Laubscher/Anelloviruses/releases/tag/Anellovirus_2023.1, release date 27 April 2023) and from a large-scale anellovirus discovery study led by Arze et al. (2021) (downloaded from https://github.com/ring-therapeutics/anellome_paper, release date 19 January 2021). This led to a final whole-genome dataset consisting of 1472 sequences (this study: 829, NCBI: 528, Tisza: 115) and an ORF1 dataset of 5940 sequences unique on the protein level.

### Phylogenetic analysis

We aligned all 5940 ORF1 protein sequences using mafft (Katoh and Standley 2013) and converted the resulting alignment to a codon alignment using pal2nal (Suyama et al. 2006). To reduce likely uninformative sites in the alignment we removed columns in which >70% of the sequences had gaps. The resulting codon alignment contained 2148 nucleotide sites.

To create the ORF1 alignment of all *Anelloviridae* genera, we first subsampled the members of each human-associated genus present in our dataset by nucleotide sequence identity using CD-HIT with a sequence threshold of 0.65 (Weizhong and Godzik 2006). The representative ORF1 sequences from the alphatorquevirus, betatorquevirus, gammatorquevirus, hetorquevirus, memtorquevirus, samektorquevirus, and lamedtorquevirus groups were combined with representative ORF1s from all other genera used for defining the family taxonomy by Varsani et al. (2021). The resulting 911 ORF1s were aligned on the protein level using mafft, converted to codon alignments with pal2nal, and columns with >70% gaps were removed as described earlier. The resulting codon alignment contained 2025 nucleotide sites.

Both phylogenies were inferred with IQ-TREE v.2.2.2.7 (Minh et al. 2020) under a GTR + F + I + R10 substitution model and node support was determined with 10 000 Ultrafast bootstrap iterations (Hoang et al. 2018).

### Recombination analysis

The 1472 whole-genome sequences (rotated to the start of ORF1 as described earlier) were aligned using mafft (–localpair option). These genomes contain extensive indel variation, resulting in many gappy regions in the alignment. Columns that consist primarily of gaps may misinform the recombination analysis, so all columns with nucleotides present for only one sequence (essentially representing insertions unique to a single genome) were removed from the alignment. The resulting alignment was examined for evidence of recombination using the Recombination Detection Program (RDP v.5.45) (Martin, et al. 2021). Modules RDP (Martin and Rybicki 2000), 3SEQ (Boni et al. 2007), GENECONV (Malla et al. 1999), Chimaera (Posada and Crandall 2001), and MaxChi (Smith 1992) were performed followed by secondary scans with methods BootScan (Martin et al. 2005) and SiScan (Gibbs et al. 2000). Sequences were assumed to be circular. Recombination events were accepted only when detected by at least by three of the methods used.

To assess potential clustering in the presence or absence of detected breakpoints across the genomes’ length (hotspots or coldspots of recombination), we performed the breakpoint distribution test (BDT) and the recombination range test (RRT) also implemented in RDP5. The type sequence was set to the TTV reference genome (NC_002076), the window size was kept at 200 nd and a total of 500 permutations were performed for each test.

### Analysis of genomic features

The aforementioned ORF1 protein alignment (*n* = 5940) was used to explore the residue composition of the ARM. The ARM region was defined as the N-terminal domain of each ORF1 until amino acid position 100 of the TTV reference genome (NC_002076) on the full ORF1 alignment. This region in the alignment matches the TTV ARM region as well as some upstream residues to capture arginine-rich regions extending further in other aligned sequences. Then, the number of arginines (R) and lysines (K) in that region was counted to represent the composition of positively charged amino acids in each virus’s putative ARM domain. Based on the distribution of R/K residue counts, it was observed that 264 ORF1s had <10 R/K residues in that part of the alignment (Figure S3). These sequences were assumed to be lacking their ARM domain and were excluded from downstream ARM composition analysis.

For the replication loop analysis, the reduced set of whole-genome sequences (*n* = 1472) was used instead. Genome sequences classified into the three main genera (alphatorquevirus, betatorquevirus, and gammatorquevirus) based on the ORF1 phylogeny were aligned to their corresponding reference using mafft (–localpair) with the—keeplength option to remove insertions relative to the reference (Katoh and Standley 2013) (alphatorquevirus: TTV NC_002076 positions 3453–3460; betatorquevirus: TTMV NC_014097 positions 2614–2621; gammatorquevirus: TTMDV NC_009225 positions 2946–2953). Members of the hetorquevirus, memtorquevirus, samektorquevirus, and lamedtorquevirus genera were aligned to the TTMV reference and manually inspected to confirm that the replication loop sequences were correctly aligned.

### Statistical analysis

The mean read depth and GC content for our assembled contigs were calculated and formatted using the Biopython (Cock et al. 2009) and pandas (The-pandas-development-team 2020) Python3 packages. All statistical comparisons between read-depth values presented in Figure S4 were performed with a Mann–Whitney *U*-test calculated using the SciPy Python3 package (Virtanen et al. 2020).

## Results

### A wide diversity of Anelloviridae in human metagenomes

**Figure 1.**
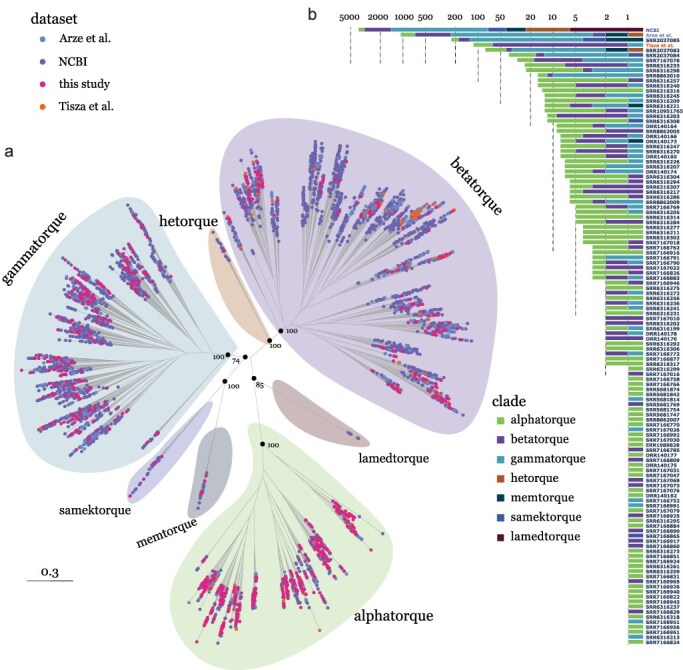
Phylogenetic and dataset distribution of all known human anelloviruses. (a) Unrooted phylogenetic tree of all available anellovirus ORF1 coding sequences. Tips are coloured by the dataset the sequences were derived from. Shading indicates the taxonomic classification of distinct clades in the tree. Ultrafast bootstrap support values are shown for key basal nodes. (b) Stacked bar plot showing the number of ORF1 sequences retrieved from each SRA sample (in this study) and from other existing datasets, separated by the sequences’ clade definition (consistent with panel a). Bars are ordered by the total number of sequences in each sample/dataset. The *x*-axis is in a base 10 logarithmic scale, absolute numbers corresponding to each axis line are shown as axis labels.

We applied Unxplore — a previously developed framework for assembling datasets of uncharacterized contigs — on 2596 publicly available human microbiome datasets and implemented an array of virus contig detection methods to look for anellovirus-related sequences (Modha 2022). In this way, we retrieved a total of 829 contigs representing distinct anellovirus genomes (220 complete and 609 near-complete) and all containing complete ORF1 genes. We combined this dataset with all published human anellovirus ORF1 coding sequences and used the resulting coding sequence alignment to infer a maximum likelihood phylogenetic tree encompassing the broadest human anellovirus genome set to date (5940 distinct ORF1 proteins; Fig. 1a). The tree formed three major clades, consistent with the three largest anellovirus genera: *Alphatorquevirus, Betatorquevirus*, and *Gammatorquevirus*. The largest numbers of ORF1 sequences assembled in this study (418) are members of the *Alphatorquevirus* genus. We further identified 228 members of the *Gammatorquevirus*, followed by 170 *Betatorquevirus* sequences, 1 *Hetorquevirus* genome, and 12 genomes of the newly defined *Memtorquevirus* and *Samektorquevirus* (6 of each genus). These proportions confirm that the first three genera are the most abundant in humans, while the latter three are generally less well represented. No members of the *Lamedtorquevirus* genus were identified in our dataset, with only six genomes having been identified previously. Reconstructing a phylogeny of a subsampled set of the human anellovirus ORF1s shown in Fig. 1a along with representatives from all other *Anelloviridae* genera shows that the human-associated anellovirus clade is monophyletic (Figure S1). The sole exception is the *Omegatorquevirus* genus represented by a single member sampled from a gorilla which is confidently nested within the human-associated clade as the closest relative to the *Hetorquevirus* and *Betatorquevirus* genera. It is unclear whether this branch represents a recent anellovirus transmission from humans to nonhuman primates, an old split consistent with the divergence of humans from gorillas, or simply human contamination of the gorilla sample. The topology of the human-associated genera was also fairly consistent in the full family tree, with the *Alphatorquevirus* genus diverging first in the clade (Figure S1). One topological difference was the placement of the *Lamedtorquevirus* genus, being sister to the *Memtorquevirus* and *Samektorquevirus* genera in the full family tree; however, the placement of this branch had lower node support compared to most basal nodes in both trees (human-associated tree: 85—Fig. 1a, family-wide tree: 88—Figure S1).

The larger proportion of *Gammatorqueviruses* (27.5% of the genomes) was the clearest discrepancy between our dataset and that of previous studies on human anelloviruses (Fig. 1b). Surprisingly, the vast majority of genomes, including more than half of our dataset’s *Gammatorqueviruses*, were found in a single SRA sample (SRR2037085, bioproject: PRJNA230363) (alphatorquevirus: 44, betatorquevirus: 55, gammatorquevirus: 124, memtorquevirus: 2, samektorquevirus: 2) associated with human oral microbiome samples (Wang et al. 2016, 2020). We further investigated the samples from this study to identify potential factors explaining this pattern. Unlike the majority of other metagenomic samples deposited by the same study, which were derived from individual patients, sample SRR2037085 contained pooled salivary samples of five individuals that were subsequently amplified for DNA viruses by purification of virus-like particles (VLP). We expect that the downstream processing steps unique to this sample are part of the reason why so many anelloviruses were retrieved from its sequencing data. VLP purification is likely a valuable step when preparing sequencing samples for the identification of anelloviruses and other small DNA viruses. Consistently, the next two samples with the largest number of assembled genomes in our dataset have undergone the same processing steps (SRR2037083 and SRR2037084; Fig. 1b). This finding may also imply that many diverse anelloviruses are missed from the metagenomic datasets when using conventional DNA sequencing protocols.

Even though this amount of, what seems to be, intra-host genetic diversity may be surprising for many viruses (especially those causing acute infection), similar findings have been shown before for anelloviruses. Specifically, Arze et al. (2021) quantified the number of distinct anellovirus lineages in individual blood donors and recipients and found up to approximately 90 and 300 lineages in a single donor and recipient, respectively. Contamination unique to the SRR2037085 sample cannot be excluded, but all genomes assembled from it are distinct and dispersed across the phylogeny of other human anelloviruses, making a single contamination source highly unlikely. Finally, to assess whether the large number of genomes was a technical artefact of our sequence assembly methodology, we reassembled the sample’s reads using a second assembly algorithm and confirmed the presence of most distinct genomes in the sample (see ‘Methods’ section).

Since the microbiome datasets come with information about the type of tissue the samples came from, we questioned whether there were specific patterns of microbiome clustering in the ORF1 phylogeny. This does not seem to be the case with no apparent clustering in the microbiome source across any of the clades in the tree (Figure S2). The majority of viruses were retrieved from blood samples (2146 out of the 2592 genomes with available microbiome information); however, this likely reflects the sampling bias towards blood transfusion patients (Arze et al. 2021), while no microbiome information was available for the majority of consensus sequences retrieved from NCBI. If anelloviruses circulate ubiquitously in the blood it is also difficult to exclude tissue contamination from circulating blood or injuries in the other sample sources assessed here (oral, pulmonary, and serum microbiomes). Based on the current metagenomic data available there is no evidence to suggest that different human tissues harbour a distinct diversity of anelloviruses.

### Extensive recombination happens nonrandomly across the genome

The wide diversity we observe across the human anelloviruses could be partly due to the viruses frequently recombining with one another. We used an array of recombination detection methods on the complete and near-complete genomes available in our datasets (*n* = 1472) to explore the extent of this process present in the evolution of human anelloviruses. We detected 345 unique recombination events, confidently inferred by at least three independent detection methods. Strikingly, 95.7% of these recombination events (330) were between genomes of the same genus (intra-genus) with only 15 (4.3%) events having probable parental sequences from different genera (inter-genus). It is worth noting that intra-genus recombination events are expected to be more difficult to detect because the parental sequences are more genetically similar to one another. Hence, we find that recombination happens extensively between closely related human anelloviruses; however, inter-genus recombinants are rarely observed in the available genomes. Many sequencing samples contained a mixture of anelloviruses from different genera (Fig. 1b), suggesting that the lack of inter-genus recombination events is not due to different genera infecting different tissues or individuals. Instead, the most likely explanation would be that inter-genus recombinants are nonreplicative due to genomic incompatibilities. Still, little is known about what cell types are infected by these viruses. Differences in cellular tropism between genera could also explain the lack of inter-genus recombination through a mechanistic rather than a selective barrier to recombination. Among the intra-genus recombination events detected, there are 241 within-alphatorquevirus, 48 within-betatorquevirus, and 41 within-gammatorquevirus. These proportions are consistent with the number of genomes from each genus included in the recombination analysis (alphatorquevirus: 803, betatorquevirus: 370, gammatorquevirus: 283). This implies that including more genomes in the analysis is likely to increase the number of detectable intra-genus recombination events.

**Figure 2.**
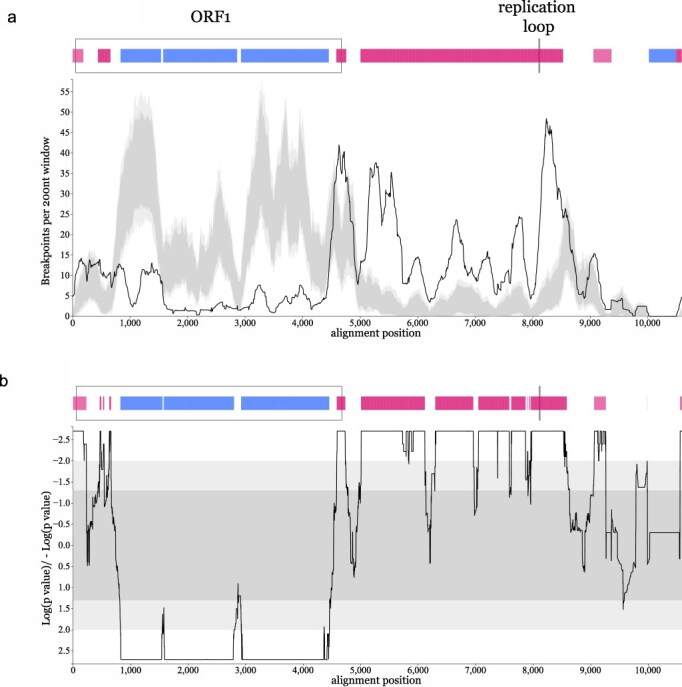
Hotspots and coldspots of recombination across human anelloviruses. Distribution of recombination hot- and coldspots across the alignment based on the RRT (a) and the BDT (b) methods. For both plots, dark grey shading represents 95% and light grey shading 99% confidence intervals of expected recombination breakpoint clustering under random recombination. Peaks above the shaded area represent recombination hotspots and drops below represent coldspots, annotated on the corresponding genome schematic above each plot by vertical pink and blue lines, respectively (99% confidence). The regions of the alignment corresponding to the TTV ORF1 coding sequence and replication loop are annotated on both genome schematics, labelled on panel (a).

We further investigated whether breakpoints of recombination were clustering in specific parts of the genome. We used the breakpoint distribution test (BDT) and the recombination range test (RRT) with the breakpoints from all 345 recombination events detected by our original analysis. These tests compare the number of breakpoints detected in each window across the genome alignment to the informative nucleotide sites in that window, determining the likelihood of detecting a breakpoint in this region of the alignment. Both tests show a very similar pattern, where most of the noncoding part of the genome has more breakpoints than expected by chance, being recombination hotspots (Fig. 2). Interestingly, the replication loop region is also determined as a hotspot of recombination, consistent with previous work suggesting that recombination in circular viral genomes tends to have one of the breakpoints near the origin of replication (Lefeuvre et al. 2009, Martin et al. 2011). In contrast, the majority of the ORF1 coding region is a clear coldspot of recombination, with very few detected breakpoints within it (Fig. 2). It is worth noting that the 5ʹ and 3ʹ ends of the coding region do contain distinct recombination hotspots. The lack of observable recombination breakpoints within ORF1, despite the extensive amount of recombination detected across the rest of the genome, may indicate that functional constraints within the gene make chimaeric ORF1s mostly deleterious for further virus replication. Another explanation might be related to breakpoints being more likely to occur closer to the origin of replication (the replication loop). These two hypotheses are not exclusive of one another. The overall pattern of recombination hot- and coldspots across the human anellovirus genomes is strikingly consistent with the results of similar analysis on felid anelloviruses (Kraberger et al. 2021b), suggesting that the mechanisms behind anellovirus recombination are conserved across the anelloviruses and not determined by the host they infect. Paired with the lack of inter-genus recombination events, we show how distinct restrictions apply to recombination across the *Anelloviridae*, both in terms of which viruses can recombine and where in the genome the recombination breakpoints can take place.

We should highlight that even though the longest genome used in our recombination analysis is 3965 nucleotides long, the alignment of all genomes has approximately 10,500 positions. This is due to the extensive indel variation present in anellovirus genomes. Even though we use conservative criteria for accepting recombination events (detected by at least three independent methods implemented in RDP5, while checking of alignment consistency), we caution that the large sequence diversity captured in our alignment could produce some false positives in the detected events.

### Genus-specific genomic features

**Figure 3.**
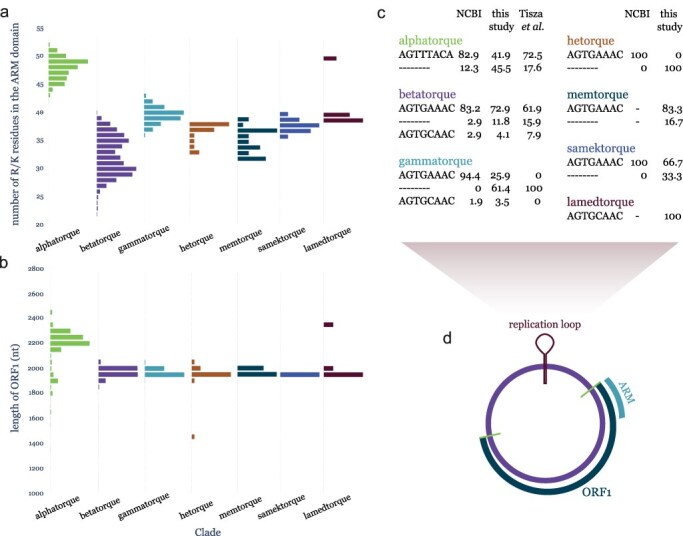
Diversity of key genomic features between human anellovirus groups. (a) Distribution of the content of arginine (R) and lysine (K) residues in the ARM domain defined for each ORF1 sequence, separated by phylogenetic group. Only sequences with a number of R/K residues over 10 are used for this analysis (see ‘Methods’ section, Supplementary Figure S3). Bar heights are normalized by the number of genomes in the largest bar. (b) Distribution of ORF1 coding sequence lengths for all ORF1s used in this study, separated by phylogenetic group. Bars represent bins of 50 nucleotides of ORF1 length each. Bar heights are normalized by the number of genomes in the largest bin. (c) Proportion of major replication loop genotypes (present in >2% of the sequences in each group) for genomes of each phylogenetic group, separated by the dataset the sequences were retrieved from. Only ORF1 coding sequences were available for the Arze et al. (2021) dataset and the majority of NCBI sequences retrieved from the SCANellome database (Laubscher et al. 2023), so they do not appear in this analysis. (d) Schematic of the anellovirus genome highlighting the relative location of the key genomic features.

The novel genomes we provide in this study also allow us to explore the diversity of key genomic features in the human anelloviruses. The N-terminal region of the anellovirus ORF1 protein is characterized by a very high content of positively charged residues (primarily arginines and lysines; Fig. 3D). This distinct protein feature is referred to as the ARM and has been shown to be involved in genome packaging by forming binding interactions with the viral DNA (Liou et al. 2022). We quantified the positively charged content of this motif for each ORF1 protein in our dataset by counting the number of arginine (R) and lysine (K) residues in the N-terminal part of the protein, corresponding to the ARM region. Based on the distribution of R/K residues present across all the ORF1 ARM regions analysed here, we proceeded to accept regions with over 10 R/K residues as complete ARMs, used for the downstream comparisons (see ‘Methods’ section, Figure S3). We find a clear separation in the number of R/K residues by the virus genus, consistent with the genome size of each taxon. *Alphatorquevirus* members had the largest number of R/Ks (mean = 48), followed by *Gammatorquevirus* (mean = 40), with the shortest taxon (*Betatorquevirus*) having the least R/K residues (mean = 32) (Fig. 3A). The three recently described genera had intermediate but varying numbers of R/K residues (memtorquevirus mean = 35, samektorquevirus mean = 38, and lamedtorquevirus mean = 41). A total of 264 ORF1 sequences (4.4% of the dataset) had less than 10 R/K residues in their N-terminal region, most having no R/Ks at all, and were deemed to have a partial or missing ARM region (Figure S3).

Another key feature of the anellovirus genome is the conserved replication loop believed to be cleaved and subsequently act as the origin of rolling-circle viral replication (Fig. 3d). We confirm that replication loop sequences are clearly conserved within anellovirus groups (Fig. 3c). As previously described, betatorqueviruses, gammatorqueviruses, and hetorqueviruses anelloviruses have the same, conserved AGTGAAAC replication loop motif; however, about 2-8% of the betatorquevirus and gammatorquevirus genomes across datasets also contain the less prevalent AGTGCAAC motif. This single nucleotide difference in this region is further reflected in the sequences of the three most recently defined genera with memtorqueviruses and samektorqueviruses having AGTGAAAC and the lamedtorquevirus having the less prevalent AGTGCAAC motif. The alphatorquevirus genus has a distinct replication loop to all other human anellovirus genera, AGTTTACA, which is consistently conserved within the genus (Fig. 3c). Along with the longer branch separating the alphatorquevirus clade from the other groups in the ORF1 phylogeny (Fig. 1a), alphatorqueviruses have likely diverged from the other groups earlier in the viruses’ evolutionary history. This is further supported by the rooting of the human-associated clade in the entire *Anelloviridae* family phylogeny (Figure S1).

In addition to the conservation of replication loop sequences, we observed that many of the genomes assembled both in this study and by previous research completely lack the replication loop (Fig. 3c). The proportion of replication loop presence varied notably across datasets, from 96.3% of the gammatorquevirus genomes retrieved from NCBI containing a complete replication loop to only 41.9% of alphatorquevirus genomes and 29.4% of gammatorquevirus genomes reported in this study having the loop sequence. The variation in the presence of a replication loop in the assembled genomes between genera and studies suggests that it cannot be fully explained by the DNA sequencing quality of each dataset or the assembly methodology in each study.

The lack of the replication loop region and the ARM-encoding part of the ORF1 gene in many of the available anellovirus genomes would imply that these genome sequences are missing key features for the viruses’ replication. If this incompleteness were an artefact of sequencing read quality, then we would expect the partial contigs to have lower overall read coverage compared to the complete ones. We compared the average read depth between contigs with and without each feature and find no difference between the depths by the presence or absence of the ARM domain (present mean depth: 1231.28, absent mean depth: 1483.76; *P*-value = .059; Figure S4A). We further inspected the per site depth of example contigs with high mean depth and absence of the ARM region. Most example contigs show uniform coverage, although few cases (e.g. contig SRR8862005_NODE_16) have local peaks in depth which may indicate misalignment or reads from different sources having been assembled in these cases (Figure S5). On the other hand, mean depth did significantly differ by the presence or absence of the replication loop region, although contigs in both categories had substantial overall read depth (present mean depth: 1938.10, absent mean depth: 703.56; *P*-value < .01; Figure S4B). The replication loop region is found in the noncoding part of the genome characterized by high GC content (Kaczorowska and Van Der Hoek 2020).

To assess the local depth in that part of the genomes, we separated our contigs into 100-bp segments with 50-bp overlaps and calculated the GC content and mean read depth for each segment. We find that segments in the top 10% of the GC content distribution in each contig have significantly lower mean read depth compared to the ones in the bottom 90% (high GC mead depth: 1222.19, normal GC mean depth: 1528.90, *P*-value <.01; Figure S4C). Hence, the absence of these key features from our genomes could partly be due to potential misassemblies. Especially in the case of the replication loop region, the GC-rich genomic context likely makes this part of the genome harder to assemble. These observations highlight the potential drawbacks of metagenomically assembling diverse viruses from publicly available short-read datasets. We propose that the use of long-read sequencing technologies in future efforts of anellovirus sequencing might largely improve the quality of genomes and elucidate the true proportion of genomes that lack their key features.

Other than the potential methodological explanation outlined earlier, there is also a biological hypothesis for the presence of intact DNA molecules of seemingly partial genomes in the samples. Villiers et al. (2011) were the first to experimentally validate the presence of diverse ‘subviral’ anellovirus genomes of varying lengths when the viruses were cultured *in vitro*. A recent study by Kaczorowska et al. (2023) monitored long-term evolution of distinct anellovirus lineages in infected individuals and further confirmed the existence of dynamic heterogeneous intra-host genomic populations. Consistent with these studies, we propose the possibility that anellovirus-infected cells can contain diverse genetic populations composed of complete, replicative genomes as well as defective viral genomes (DVGs), some of which may be nonreplicative. Although the term DVGs is common in the literature, we prefer the use of non-standard viral genomes (nsVGs), since little is known about the function of these partial anellovirus genomes, which could contribute to the intra-host viral community rather than being truly ‘defective’. In summary, we expect that some of the contigs in our dataset lacking key features may reflect nsVGs that comprise the overall genomic heterogeneity in each sample.

## Discussion

In this study, we provide 829 novel anellovirus genomes retrieved from more than 2000 publicly available human metagenomic samples. These viruses are widely distributed across the known diversity of human anelloviruses, reflecting the heterogeneity of genomes within the analysed metagenomes. We use all known human anellovirus genomes to study the patterns of recombination in this virus group. Our analysis reveals that, even though recombination takes place extensively between genomes, there are restrictions pertaining to the mechanism of anellovirus recombination. Specifically, the vast majority of detected recombination events are between viruses belonging to the same phylogenetic clade, while breakpoints of recombination rarely occur within the ORF1 coding region. The absence of otherwise highly conserved genomic features necessary for virus replication in some of the assembled genomes leads us to hypothesize that anellovirus populations potentially include substantial proportions of nsVGs. We expect that this intra-host heterogeneity is a major driver of the extensive recombination patterns we describe in this manuscript. The genomic diversity we present here is likely only the tip of the iceberg that is the human anellovirus diversity. At the same time, we caution that potential artefacts stemming from the metagenomic approach may also explain the incomplete nature of these genomes and future research using long-read sequencing could shed light to the hypotheses posed here. Anelloviruses have recently shown promise to be used as biomarkers of immunosuppression in patients (Castain et al. 2024) as well as vectors for gene therapy (Prince et al. 2024). Better describing the diversity of these ubiquitous commensals can improve the development of medical applications, but also reveal more on how circular DNA viruses utilize features like recombination and nsVGs during their evolution.

## Supplementary Material

veaf002_Supp

## Data Availability

Assembled contigs are available in the Third Party Annotation Section of the DDBJ/ENA/GenBank databases under the accession numbers TPA: BK068993-BK069821. All raw data, assembled contigs, alignments, phylogenies, and associated codes are available at https://doi.org/10.5281/zenodo.14408301. Data can also be accessed from the associated project GitHub repository https://github.com/spyros-lytras/anellovirus-diversity.

## References

[R1] Abe K, Inami T, Ishikawa K et al. TT virus infection in nonhuman primates and characterization of the viral genome: identification of simian tt virus isolates. *J Virol* 2000;74:1549. doi: 10.1128/JVI.74.31549-1553.2000 PMC111492 10627568

[R2] Arze CA, Springer S, Dudas G et al. Global genome analysis reveals a vast and dynamic anellovirus landscape within the human virome. *Cell Host Microbe* 2021;29:1305–1315.e6. doi: 10.1016/J.CHOM.2021.07.001 34320399

[R3] Boni MF, Posada D, Feldman MW. An exact nonparametric method for inferring mosaic structure in sequence triplets. *Genetics* 2007;176:1035–47. doi: 10.1534/genetics.106.068874 17409078 PMC1894573

[R4] Buchfink B, Reuter K, Drost H-G. Sensitive protein alignments at tree-of-life scale using DIAMOND. *Nat Methods* 2021;18:366–68. doi: 10.1038/s41592-021-01101-x 33828273 PMC8026399

[R5] Butkovic A, Kraberger S, Smeele Z et al. Evolution of anelloviruses from a circovirus-like ancestor through gradual augmentation of the jelly-roll capsid protein. *Virus Evolut* 2023;9:vead035. doi: 10.1093/ve/vead035 PMC10266747 37325085

[R6] Camacho C, Coulouris G, Avagyan V et al. BLAST plus: architecture and applications. *BMC Bioinf* 2009;10:9. doi: 10.1186/1471-2105-10-421 PMC2803857 20003500

[R7] Castain L, Petrier M, Bulteau S et al. Association of dynamics of anellovirus loads with hospital-acquired pneumonia in brain-injured patients during the intensive care unit stay. *J Infect Dis* 2024;230:1139–46. doi: 10.1093/infdis/jiae110 38428995

[R8] Cebriá-Mendoza M, Arbona C, Larrea L et al.. Deep viral blood metagenomics reveals extensive anellovirus diversity in healthy humans. *Sci Rep* 2021;11:1–11. doi: 10.1038/s41598-021-86427-4 33767340 PMC7994813

[R9] Cock PJA, Antao T, Chang JT et al. Biopy- thon: Freely available python tools for computational molecular biology and bioinformatics. *Bioinformatics* 2009;25:1422–23. doi: 10.1093/bioinformatics/btp163 19304878 PMC2682512

[R10] Cossart Y . TTV - A virus searching for a disease. *J Clin Virol* 2000;17:1–3. doi: 10.1016/S1386-6532(00)00078-0 10814932

[R11] Dinghua L, Liu C-M, Luo R et al. MEGAHIT: an ultra-fast single-node solution for large and complex metagenomics assembly via succinct de Bruijn graph. *Bioinformatics* 2015;31:1674–76. doi: 10.1093/bioinformatics/btv033 25609793

[R12] Edgar RC, Taylor B, Lin V et al. Petabase- scale sequence alignment catalyses viral discovery. *Nature* 2022;602:142–47. doi: 10.1038/s41586-021-04332-2 35082445

[R13] Edwards RA, Rohwer F. Viral metagenomics. *Nat Rev Microbiol* 2005;3:504–10. doi: 10.1038/nrmicro1163 15886693

[R14] Fahsbender E, Burns JM, Kim S et al. Diverse and highly recombinant anelloviruses associated with Weddell seals in Antarctica. *Virus Evolut* 2017;3:17. doi: 10.1093/ve/vex017 PMC5518176 28744371

[R15] Gibbs MJ, Armstrong JS, Gibbs AJ. Sister-scanning: a Monte Carlo procedure for assessing signals in rebombinant sequences. *Bioinformatics* 2000;16:573–82. doi: 10.1093/bioinformatics/16.7.573 11038328

[R16] Guo J, Bolduc B, Zayed AA et al. VirSorter2: a multi-classifier, expert-guided approach to detect diverse DNA and RNA viruses. *Microbiome* 2021;9:37. doi: 10.1186/s40168-020-00990-y PMC7852108 33522966

[R17] Hoang DT, Chernomor O, Von Haeseler A et al. UFBoot2: improving the ultrafast bootstrap approximation. *Mol Biol Evolut* 2018;35:518–22. doi: 10.1093/molbev/msx281 PMC5850222 29077904

[R18] Huerta-Cepas J, Serra F, Bork P. ETE 3: Reconstruction, analysis, and visualization of phylogenomic data. *Mol Biol Evolut* 2016;33:1635–38. doi: 10.1093/molbev/msw046 PMC4868116 26921390

[R19] Itoh Y, Takahashi M, Fukuda M et al. Visualization of TT virus particles recovered from the sera and feces of infected humans. *Biochem Biophys Res Commun* 2000;279:718–24. doi: 10.1006/BBRC.2000.4013 11118351

[R20] Kaczorowska J, Timmerman AL, Deijs M et al. Anellovirus evolution during long-term chronic infection. *Virus Evolut* 2023;9:vead001. doi: 10.1093/ve/vead001 PMC9885978 36726484

[R21] Kaczorowska J, Van Der Hoek L. Human anelloviruses: Diverse, omnipresent and commensal members of the virome. *FEMS Microbiol Rev* 2020;44:305–13. doi: 10.1093/femsre/fuaa007 32188999 PMC7326371

[R22] Katoh K, Standley DM. MAFFT multiple sequence alignment software version 7: improvements in performance and usability. *Mol Biol Evolut* 2013;30:772–80. doi: 10.1093/molbev/mst010 PMC3603318 23329690

[R23] Koonin EV, Dolja VV, Krupovic M. The healthy human virome: from virus–host symbiosis to disease. *Curr Opin Virol* 2021;47:86–94. doi: 10.1016/J.COVIRO.2021.02.002 33652230

[R24] Kraberger S, Opriessnig T, Celer V et al. Taxonomic updates for the genus gyrovirus (family Anelloviridae): recognition of several new members and establishment of species demarcation criteria. *Arch Virol* 2021a;166:2937–42. doi: 10.1007/S00705-021-05194-9 34347169

[R25] Kraberger S, Serieys LEK, Richet C et al. Complex evolutionary history of felid anelloviruses. *Virology* 2021b;562:176–89. doi: 10.1016/J.VIROL.2021.07.013 34364185

[R26] Laubscher F, Hartley M-A, Kaiser L et al. Genomic diversity of torque teno virus in blood samples from febrile paediatric outpatients in Tanzania: a descriptive cohort study. *Viruses* 2022;14:1612. doi: 10.3390/v14081612 PMC9330782 35893678

[R27] Laubscher F, Kaiser L, Cordey S. SCANellome: analysis of the genomic diversity of human and non-human primate anelloviruses from metagenomics data. *Viruses* 2023;15:1575. doi: 10.3390/v15071575 PMC10384568 37515261

[R28] Lefeuvre P, Lett J-M, Varsani A et al. Widely conserved recombination patterns among single-stranded dna viruses. *J Virol* 2009;83:2697–707. doi: 10.1128/JVI.02152-08 19116260 PMC2648288

[R29] Liou S-H, Cohen N, Zhang Y et al. (2022). Anellovirus structure reveals a mechanism for immune evasion. *BioRxiv*. 498313. doi: 10.1101/2022.07.01.

[R30] Malla P, Sawyer S, Fauquet CM. Possible emergence of new geminiviruses by frequent recombination. *Virology* 1999;265:218–25. doi: 10.1006/viro.1999.0056 10600594

[R31] Martin DP, Biagini P, Lefeuvre P et al. Recombination in eukaryotic single stranded DNA viruses. *Viruses* 2011;3:1699–738. doi: 10.3390/v3091699 21994803 PMC3187698

[R32] Martin DP, Posada D, Crandall KA et al. A modified bootscan algorithm for automated identification of recombinant sequences and recombination breakpoints. *AIDS Res Hum Retroviruses* 2005;21:98–102. doi: 10.1089/aid.2005.21.98 15665649

[R33] Martin DP, Rybicki E. RDP: detection of recombination amongst aligned sequences. *Bioinform Applications Note* 2000;16:562–63. doi: 10.1093/bioinformatics/16.6.562 10980155

[R34] Martin DP, Varsani A, Roumagnac P et al. RDP5: a computer program for analysing recombination in, and removing signals of recombination from, nucleotide sequence datasets. *Virus Evolut* 2021;7:87. doi: 10.1093/ve/veaa087 PMC8062008 33936774

[R35] Minh BQ, Schmidt HA, Chernomor O et al. IQ-TREE 2: new models and efficient methods for phylogenetic inference in the genomic era. *Mol Biol Evolut* 2020;37:1530–34. doi: 10.1093/MOLBEV/MSAA015 PMC7182206 32011700

[R36] Miyata H, Tsunoda H, Kazi A et al. Identification of a novel GC-rich 113-nucleotide region to complete the circular, single-stranded DNA genome of TT virus, the first human circovirus. *J Virol* 1999;73:3582–86. doi: 10.1128/jvi.73.5.3582-3586.1999 10196248 PMC104131

[R37] Modha S . Sequence data mining and characterisation of unclassified microbial diversity. PhD thesis. University of Glasgow, pp. 1–236, 2022

[R38] Modha S, Robertson DL, Hughes J et al. Quantifying and cataloguing unknown sequences within human microbiomes. *mSystems* 2022;7:e01468–21. doi: 10.1128/MSYSTEMS.01468-21 35258340 PMC9052204

[R39] Moustafa A, Xie C, Kirkness E et al. The blood DNA virome in 8,000 humans. *PLOS Pathogens* 2017;13.3:e1006292. doi: 10.1371/journal.ppat.1006292 PMC5378407 28328962

[R40] Nayfach S, Roux S, Seshadri R et al. A genomic catalog of earth’s microbiomes. *Nat Biotechnol* 2021;39:499–509. doi: 10.1038/s41587-020-0718-6 33169036 PMC8041624

[R41] Nishizawa T, Okamoto H, Konishi K et al. A novel DNA virus (TTV) associated with elevated transaminase levels in posttransfusion hepatitis of unknown etiology. *Biochem Biophys Res Commun* 1997;241:92–97. doi: 10.1006/bbrc.1997.7765 9405239

[R42] Okamoto H, Nishizawa T, Takahashi M et al. Genomic and evolutionary characterization of TT virus (TTV) in tupaias and comparison with species-specific TTVs in humans and non-human primates. *J Gen Virol* 2001;82:2041–50. doi: 10.1099/0022-1317-82-9-2041 11514713

[R43] Okamoto H, Nishizawa T, Tawara A et al. Species-specific TT viruses in humans and nonhuman primates and their phylogenetic relatedness. *Virology* 2000;277:368–78. doi: 10.1006/VIRO.2000.0588 11080484

[R44] Posada D, Crandall KA. Evaluation of methods for detecting recombination from DNA sequences: computer simulations. *Proc Natl Acad Sci* 2001;98:13757–62. doi: 10.1073/pnas.241370698 11717435 PMC61114

[R45] Prince C, Bounoutas G, Zhou B et al. (2024). A novel functional gene delivery platform based on a commensal human anellovirus demonstrates transduction in multiple tissue types. *BioRxiv*10.1016/j.omtm.2025.101597 PMC12538900 41127004

[R46] Ren J, Song K, Deng C et al. Identifying viruses from metagenomic data using deep learning. *Quant Biol* 2020;8:64–77. doi: 10.1007/s40484-019-0187-4 34084563 PMC8172088

[R47] Rice P, Longden I, Bleasby A. EMBOSS: the European molecular biology open software suite. *Trends Genet* 2000;16:276–77. doi: 10.1016/S0168-9525(00)02024-2 10827456

[R48] Seemann T . Prokka: rapid prokaryotic genome annotation. *Bioinformatics* 2014;30:2068–69. doi: 10.1093/bioinformatics/btu153 24642063

[R49] Simmonds P, Davidson F, Lycett C et al. Detection of a novel DNA virus (TTV) in blood donors and blood products. *Lancet* 1998;352:191–95. doi: 10.1016/S0140-6736(98)03056-6 9683208

[R50] Smith JM . Analyzing the mosaic structure of genes. *J Mol Evolut* 1992;34:126–29. doi: 10.1007/BF00182389 1556748

[R51] Steinegger M, Söding J. MMseqs2 enables sensitive protein sequence searching for the analysis of massive data sets. *Nat Biotechnol* 2017;35:1026–28. doi: 10.1038/nbt.3988 29035372

[R52] Suyama M, Torrents D, Bork P. PAL2NAL: robust conversion of protein sequence alignments into the corresponding codon alignments. *Nucleic Acids Res* 2006;34:W609–612. doi: 10.1093/nar/gkl315 16845082 PMC1538804

[R53] Taxonomy of Viruses, International Committee on (2011). Anelloviridae.

[R54] The-pandas-development-team . pandas-dev/pandas: Pandas. Version latest, 2020.

[R55] Tisza MJ, Pastrana DV, Welch NL et al. Discovery of several thousand highly diverse circular DNA viruses. *eLife* 2020;9:e51971. doi: 10.7554/eLife.51971 PMC7000223 32014111

[R56] Varsani A, Opriessnig T, Celer V et al. Taxonomic update for mammalian anelloviruses (family Anelloviridae). *Arch Virol* 2021;166:2943–53. doi: 10.1007/S00705-021-05192-X 34383165

[R57] Vasilyev EV, Trofimov DY, Tonevitsky AG et al. Torque teno virus (TTV) distribution in healthy Russian population. *Virol J* 2009;6:134. doi: 10.1186/1743-422X-6-134 PMC2745379 19735552

[R58] Villiers E-MD, Borkosky SS, Kimmel R et al. The diversity of torque teno viruses: in vitro replication leads to the formation of additional replication- competent subviral molecules. *J Virol* 2011;85:7284–95. doi: 10.1128/jvi.02472-10 21593173 PMC3126604

[R59] Virtanen P, Gommers R, Oliphant TE et al. SciPy 1.0: Fundamental algorithms for scientific computing in python. *Nat Methods* 2020;17:261–72. doi: 10.1038/s41592-019-0686-2 32015543 PMC7056644

[R60] Wang J, Gao Y, Zhao F. Phage-bacteria interaction network in human oral microbiome. *Environ Microbiol* 2016;18:2143–58. doi: 10.1111/1462-2920.12923 26036920

[R61] Wang J, Jia Z, Zhang B et al. Tracing the accumulation of in vivo human oral microbiota elucidates microbial community dynamics at the gateway to the GI tract. *Gut* 2020;69:1355–56. doi: 10.1136/GUTJNL-2019-318977 31227588 PMC7306975

[R62] Weizhong L, Godzik A. Cd-hit: a fast program for clustering and comparing large sets of protein or nucleotide sequences. *Bioinformatics* 2006;22:1658–59. doi: 10.1093/bioinformatics/btl158 16731699

[R63] Wolf YI, Silas S, Wang Y et al. Doubling of the known set of RNA viruses by metagenomic analysis of an aquatic virome. *Nat Microbiol* 2020;5:1262–70. doi: 10.1038/s41564-020-0755-4 32690954 PMC7508674

[R64] Worobey M . Extensive homologous recombination among widely divergent TT viruses. *J Virol* 2000;74:7666–70. doi: 10.1128/jvi.74.16.7666-7670.2000 10906223 PMC112290

