# Supplementary material for: Expanding the genomic diversity of human anelloviruses
Source: Virus Evol. 2025 Jan 7;11(1):veaf002. doi: 10.1093/ve/veaf002 (PMC11749082; doi:10.1093/ve/veaf002)
Supplement: veaf002_Supp [file veaf002_supp.zip › suppl_data/figS4_r1_fn.pdf]

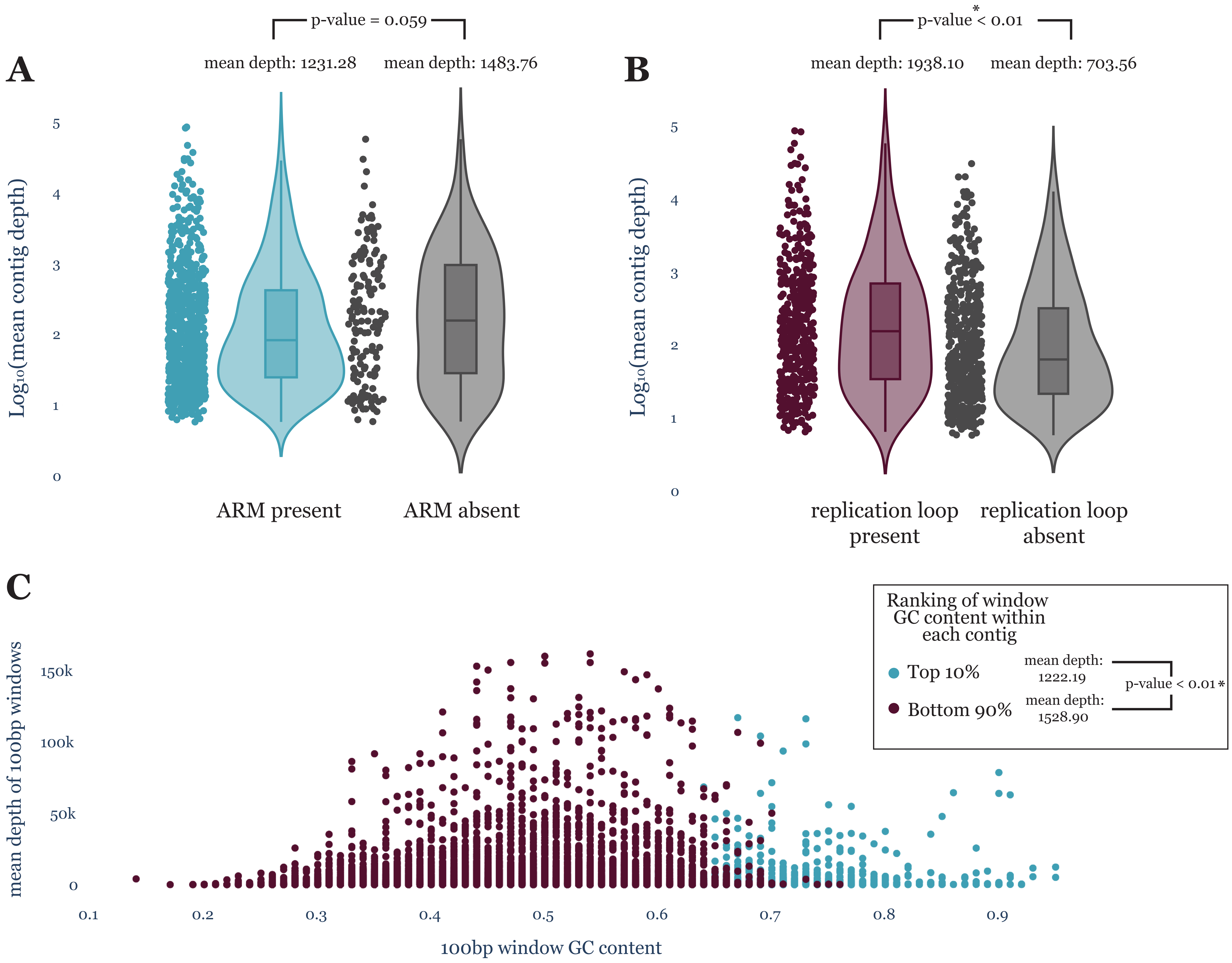

A
p-value = 0.059
mean depth: 1231.28
mean depth: 1483.76
Log10(mean contig depth)
0
1
2
3
4
5
ARM present
ARM absent
B
p-value < 0.01*
mean depth: 1938.10
mean depth: 703.56
Log10(mean contig depth)
0
1
2
3
4
5
replication loop present
replication loop absent
C
Ranking of window GC content within each contig
Top 10%
Bottom 90%
mean depth: 1222.19
mean depth: 1528.90
p-value < 0.01*
mean depth of 100bp windows
0
50k
100k
150k
0.1
0.2
0.3
0.4
0.5
0.6
0.7
0.8
0.9
100bp window GC content
