# Supplementary figures and images for: Expanding the genomic diversity of human anelloviruses
Source: Virus Evol. 2025 Jan 7;11(1):veaf002. doi: 10.1093/ve/veaf002 (PMC11749082; doi:10.1093/ve/veaf002)

Human-associated  
Anelloviruses

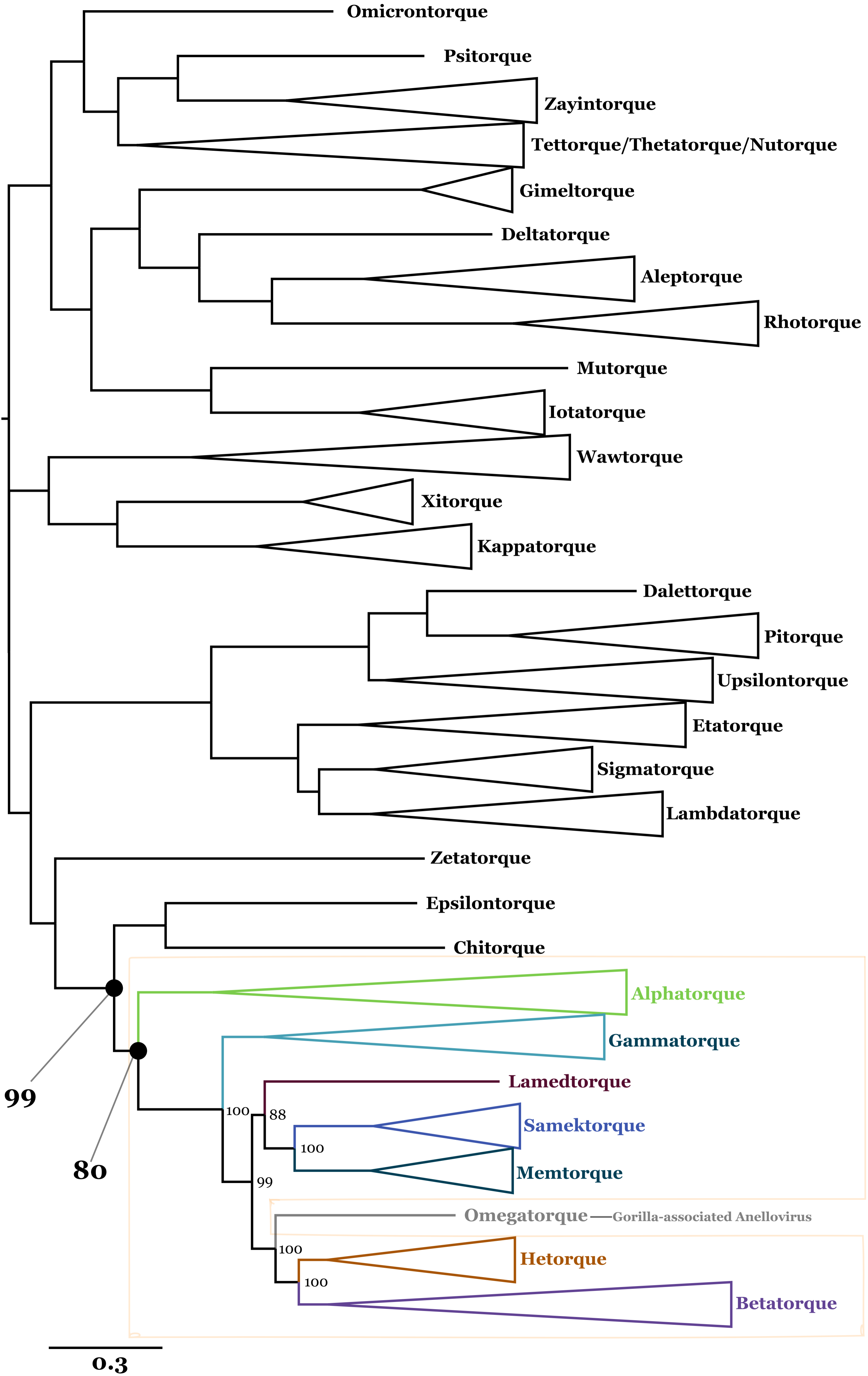

Supplement: veaf002_Supp [file veaf002_supp.zip › suppl_data/figS1_r1_fn.pdf]

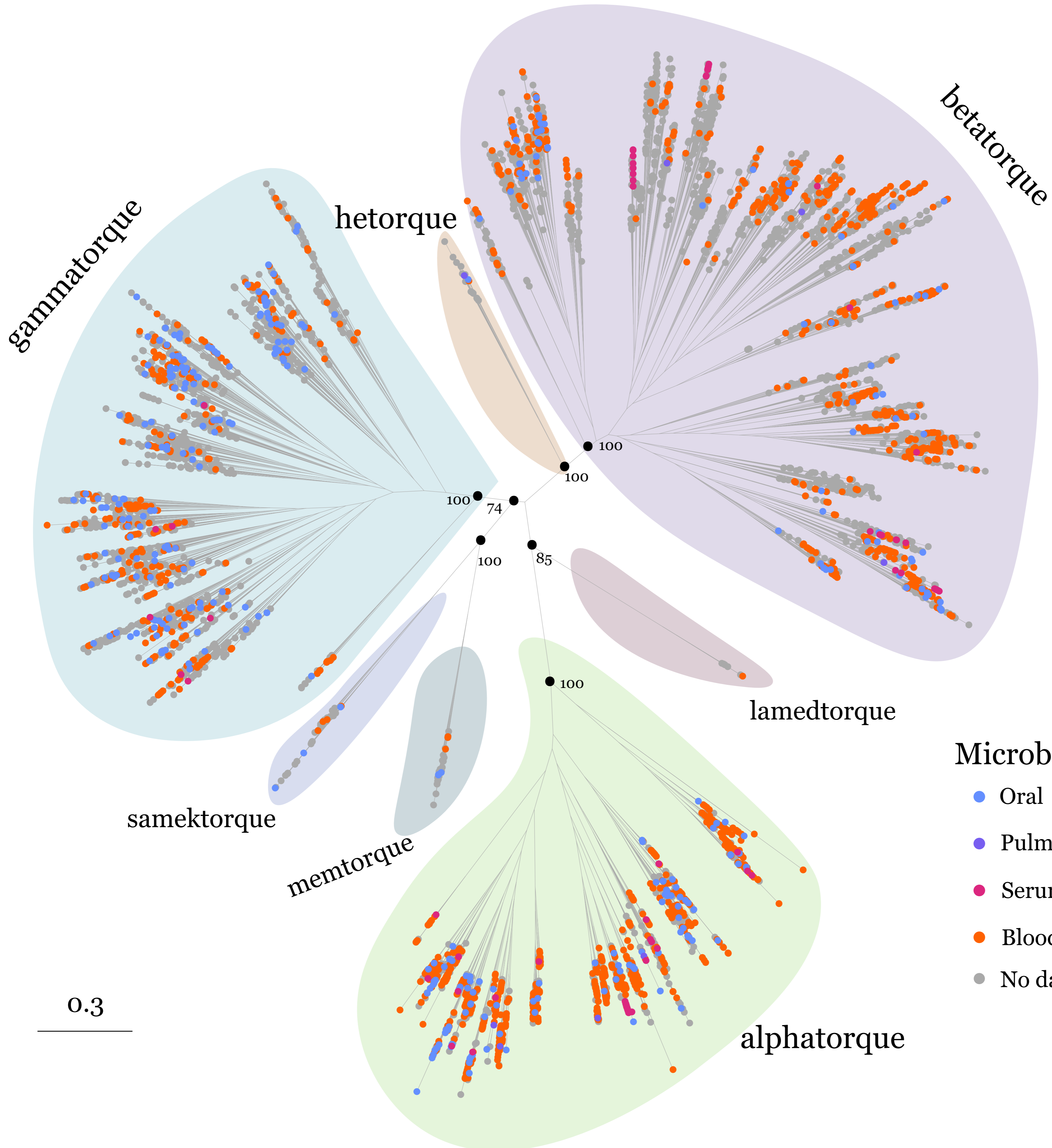

- Microbiome
- Oral
  - Pulmonary system
  - Serum
  - Blood
  - No data

Supplement: veaf002_Supp [file veaf002_supp.zip › suppl_data/figS2_r1_fn.pdf]

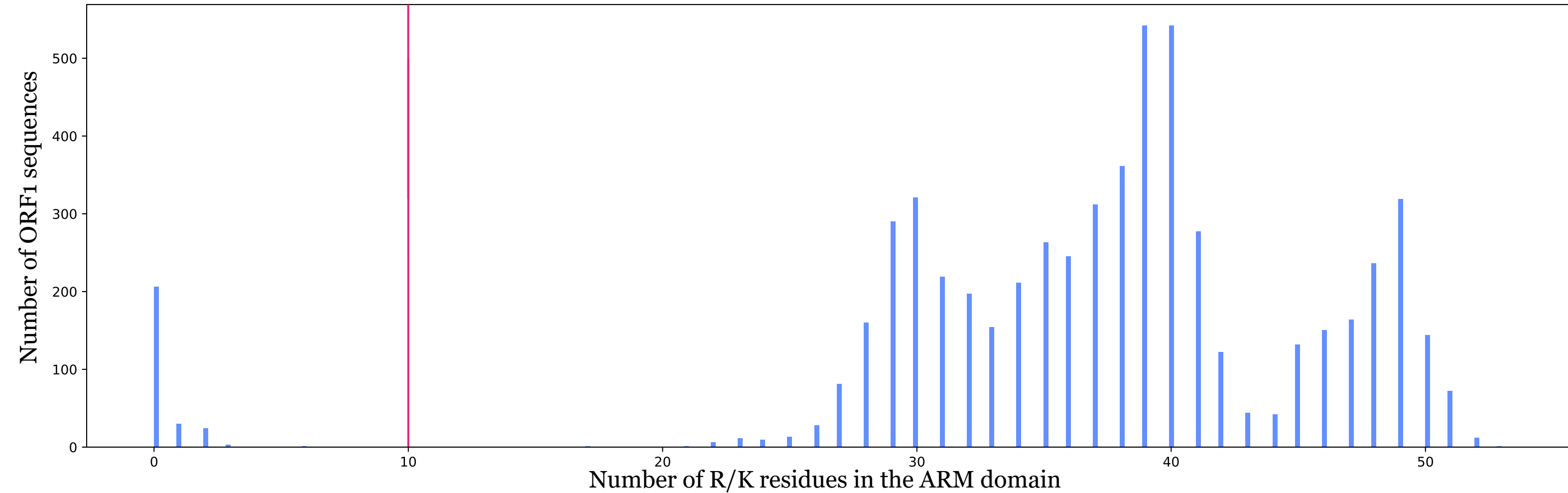

Supplement: veaf002_Supp [file veaf002_supp.zip › suppl_data/figS3_r1_fn.pdf]

Sample ID=SRR8862005

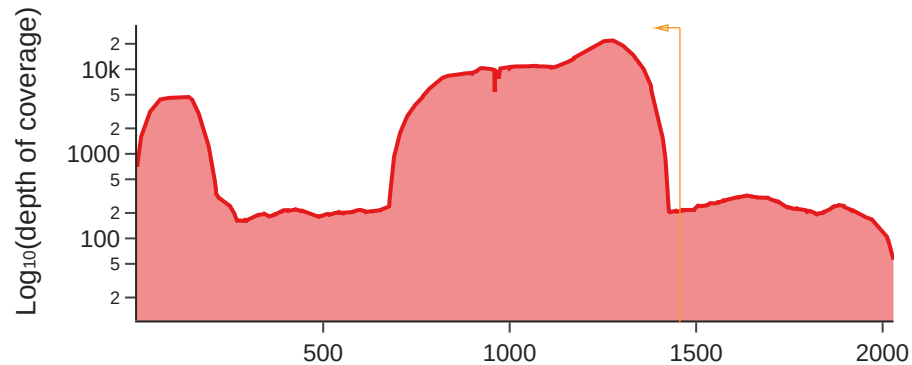

Sample ID=SRR6316233

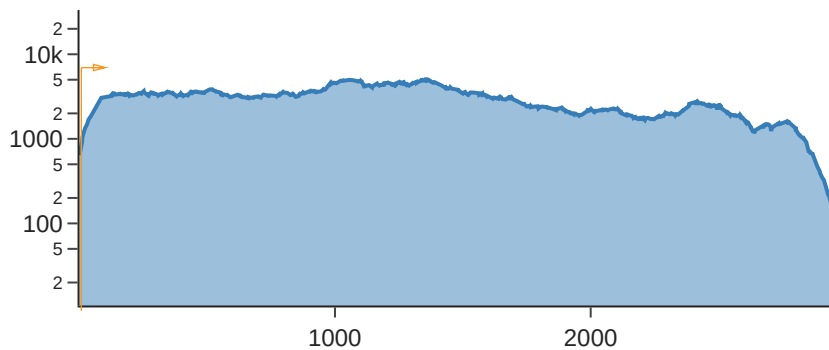

Sample ID=SRR2037085

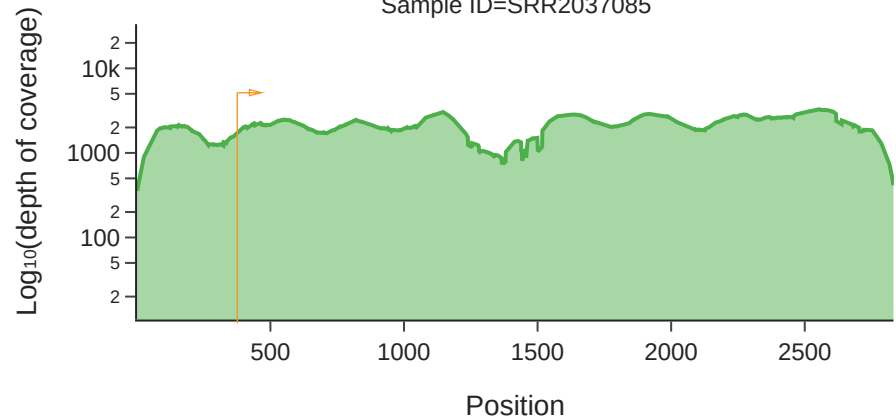

Sample ID=SRR6316298

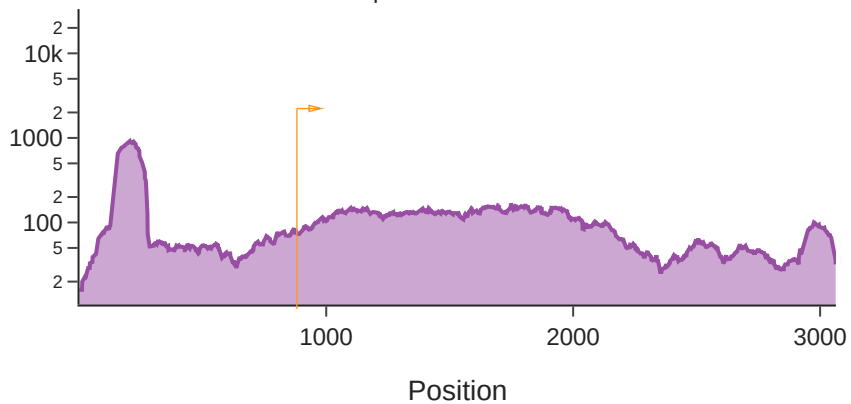

Supplement: veaf002_Supp [file veaf002_supp.zip › suppl_data/figS5_r1_fn.pdf]
